# School prevention of non-consensual sexting among middle adolescents: Does sexual preoccupation awareness matter?

**DOI:** 10.3389/fpsyg.2024.1384620

**Published:** 2024-05-23

**Authors:** Thanos Touloupis

**Affiliations:** Department of Primary Education, University of the Aegean, Rhodes, Greece

**Keywords:** non-consensual sexting, prevention, sexual preoccupation, middle adolescents, junior high schools

## Abstract

Although non-consensual sexting seems to concern not only adults but also middle adolescent students, with detrimental consequences on their well-being, the related research-based effective school prevention programs are almost absent. Furthermore, there is an unanswered research question as to whether sex-related issues, such as sexual preoccupation, matters in adolescents’ non-consensual sexting and its prevention. The present study investigated the effectiveness of a school-based intervention against non-consensual sexting among middle adolescents, based on the European funded program TABBY (Threat Assessment of Bullying Behavior in Youth). Furthermore, the predictive role of sexual preoccupation was examined. Overall, 280 Greek students from randomly selected junior high school responded to self-report scales on non-consensual sexting and sexual preoccupation. Based on an experimental longitudinal research design, only the experimental (*N* = 131), but not the control group of students (*N* = 149), participated in the intervention. To test the effectiveness of the intervention, both student groups completed the scales before (1st phase), immediately after (2nd phase), and six months after the intervention (3rd phase), which was implemented by trained teachers. The results showed both, the short-term and long-term effectiveness of the intervention. Also, the study highlighted the significant contribution of sexual preoccupation awareness in reducing non-consensual sexting, especially immediately after and six months after the intervention. The study implies the necessity to adapt existed prevention programs to the specific parameters of non-consensual sexting, integrating at the same time sexual preoccupation awareness activities to better address this issue.

## 1 Introduction

The extensive use of digital technologies by students during and after the end of the COVID-19 pandemic has intensified the manifestation of online risk behaviors, especially in middle adolescence (13–15 years old) during which social media use usually peaks (Schønning et al., 2020). Except for cyberbullying, which is widely investigated among adolescents (Brochado et al., 2017; Zhu et al., 2021), sexting behaviors have emerged as another more specific pattern of online risk behaviors intertwined with individuals’ sexuality, which is usually intense during adolescence (Lightfoot et al., 2022).

Sexting concerns the exchange of sexually explicit messages/images (sexts) via the internet/mobile phones (Bianchi et al., 2019). Several adolescents report that sexting can be experienced as a safer form of communication, compared to having a real romantic relationship, or that the exchange of sexts is considered to be normal between two adolescents who are in some form of a romantic relationship (Lenhart, 2009). In this context, we talk about consensual sexting, which refers to the exchange of messages between people with mutual respect and consent, without exerting pressure and without sharing these messages with third parties. On the other hand, non-consensual sexting refers to all those cases where sexual material is used and trafficked in an unfair and irresponsible way (e.g., non-consensual sending, receiving, and third-party forwarding) involving elements of harassment and/or violence (Ringrose et al., 2012).

Recent systematic reviews (e.g., Paradiso et al., 2023) have pointed out that sexting has become a phenomenon with global dimensions and several psychological, relational, and social factors have been associated with its manifestation. However, only very few studies focus on sexting in adolescence (under 18 years old), showing however that almost 15% of them report sending sexts, almost 28% have received sexts, and approximately 9% have forwarded sexts to third parties without consent (Madigan et al., 2018; Barroso et al., 2021). The above findings show clearly that sexting concerns, not only adults, but also younger age groups. Nevertheless, it should be highlighted that the above studies tend to investigate sexting as a unified phenomenon, without always emphasizing its non-consensual dimensions. Therefore, it is sometimes ambiguous if and to what extent adolescents are put at risk due to their involvement in sexting behaviors and therefore if school prevention actions are required. In contrast, the present study focuses exclusively on non-consensual sexting, which reflects a less secure and responsible online sexual behavior among adolescents. In this way, school stakeholders can be clearly aware if sexting constitutes a really risk condition for the under-investigated age group of adolescents. The latter is of crucial importance considering that sexting has been associated with adolescents’ deviant/illegal behaviors, anxious/depressive symptoms, substance abuse, and interpersonal problems (Mori et al., 2019; Van Ouytsel et al., 2020; Lee and Darcy, 2021). The present study could highlight more clearly the necessity to prevent sexting from the onset of adolescence, during which individuals face different developmental challenges, such as experimentation and sex identity research (Lightfoot et al., 2022), and therefore they could be considered a vulnerable group for engaging in online risk sexual behaviors (sexting).

International studies have examined the effectiveness of prevention programs against cyberbullying, which is also taken place via the internet/mobile phones (Buils et al., 2020; Gabrielli et al., 2021; Touloupis and Athanasiades, 2022). The effectiveness of these programs is usually concluded by comparing students’ cyberbullying rates before and after the implementation of the related intervention (Buils et al., 2020; Gabrielli et al., 2021; Touloupis and Athanasiades, 2022). In international literature a “before” (pre-test) and “after” (post-test) assessment, based on self-reports, is also used to compare and measure effects of a prevention program on other risky behaviors in a physical context [International Alliance for Responsible Drinking (IARD)—Toolkit, 2015]. This impact-based type of evaluation is also considered by systematic and meta-analytical reviews on related prevention programs (Tanrikulu, 2018; Gaffney et al., 2019). In contrast to cyberbullying prevention programs, preventing sexting should presuppose an intervention that is more focused on the sexual nature of sexting behaviors, compared to prevention programs that adopt more general guidelines about safe online culture. According to the author’s knowledge, to date almost no study has emphasized exclusively the effectiveness of a prevention program against sexting, which has been reported as a risk factor for high-risk real sexual behavior (Benotsch et al., 2013). The only available findings concern mainly literature reviews on general guidelines regarding preventing sexting (Hinduja and Patchin, 2010; Van Ouytsel et al., 2014; Bhat, 2018; Ojeda Pérez and Rey Alamillo, 2021). Among others, these guidelines emphasize, not only lectures and presentations about the nature of sexting behaviors and its associated risks, but also audio-visual material, peer group experiential collaborative activities that promote sexual ethics, sensitization about gender-based sexual stereotypes, and development of school rules and protocols against sexting (Hinduja and Patchin, 2010; Van Ouytsel et al., 2014; Bhat, 2018; Ojeda Pérez and Rey Alamillo, 2021). Also, very limited studies describe related projects/actions implemented in the school context, however without information about their effectiveness against sexting. For example, Kopecký (2012) mention the “Webrangers” education project, which included students’ training in online risk behaviors (e.g., sexting, cyberbullying) and subsequently students’ engagement in workshops/activities aimed at raising awareness of the school community. Also, Ferrari et al. (2016), present the “Image.me” research project about sexting prevention, which among others utilizes theatrical activities to promote students’ collaborative skills and sensitization regarding sexting behaviors. Nevertheless, in both cases, no reports regarding the short-term or long-term effectiveness of these projects are available. Consequently, research-based findings regarding the effectiveness of a specific prevention program against sexting among adolescents are necessary.

Discussing about prevention, the most important parameter that contributes to the effective implementation of prevention actions are the factors that trigger a deviant/risky behavior either in a physical or electronic context. Therefore, it is considered important to identify those factors that can act protectively against sexting. To date, related studies concern primarily adolescents’ personality traits (e.g., narcissism), or attitudes and intentions towards sexting (Alonso and Romero, 2019; Hernández et al., 2021; Morelli et al., 2021). On the contrary, minimal research attention has received the predictive role of adolescents’ sex-related psychological mechanisms, such as sex preoccupation, in their engagement in sexting behaviors (Clancy et al., 2021). Sexual preoccupation concerns individuals’ increased interest in sexual fantasies, thoughts, or activities (Clancy et al., 2021). Considering that one of the most common developmental challenges that individuals usually experience during adolescence is the formation of sex identity (Lightfoot et al., 2022), it is important to further investigate not only the predictive role of sexual preoccupation in sexting behaviors but also whether awareness actions about sexual preoccupation could be incorporated into a broader prevention project against non-consensual sexting behaviors. Based on the author’s search in widely used scientific research databases (e.g., Scopus, Google Scholar, Science Direct, Web of Science, PubMed, JSTOR) and using alternative combinations of related keywords (e.g., sexting and sexual preoccupation, prevention of sexting and sexual preoccupation, cyberbullying and sexual preoccupation, prevention of cyberbullying and sexual preoccupation) in both Greek and international literature (journal articles, books/chapters, conference papers/abstracts) no related international findings are identified to date.

Therefore, examining the effectiveness of a prevention program against adolescents’ non-consensual sexting, enriched with awareness activities about sexual preoccupation could fill an important literature gap. As the present study was conducted in Greece, the TABBY program (Threat Assessment of Bullying Behavior in Youth) was utilized, as it has already been implemented successfully in Greek high schools and elementary schools to prevent and reduce cyberbullying among students (Athanasiades et al., 2015; Touloupis and Athanasiades, 2022). The TABBY program, which constitutes a funded program of European standards [Threat Assessment of Bullying Behavior in Youth (TABBY), n.d.], although initially designed for cyberbullying behaviors, it was chosen for the prevention of sexting as it meets effectively most of the guidelines described earlier for the prevention of sexting (e.g., presentations/lectures, audio-visual material, peer group experiential collaborative activities) (Athanasiades et al., 2015; Touloupis and Athanasiades, 2022). However, for the needs of the present study, out of the four audio-visual materials (videos) of the program, emphasis was given only to one entitled “Joke or Serious Crime,” which negotiates a behavior indicative of sexting (non-consensual forwarding/posting of semi-naked pictures of a female student). The other three videos were not utilized since they concerned common forms of cyberbullying (e.g., sending offensive instant messages without sexual content) and not specific behaviors indicative on non-consensual sexting. Furthermore, the intervention included presentations/lectures, experiential activities and the development of school rules related to the difference between consensual and non-consensual sexting, the behaviors that are indicative of non-consensual sexting and social–emotional consequences of sexting on individual’s life, as well as the gender-based sexual stereotypes and the sexual ethical behaviors. Finally, the intervention was enhanced with more peer group collaborative activities emphasizing healthy ways of processing and externalizing sexual thoughts or related emotions. In this way, the issue of sexual preoccupation could be processed and discussed within a positive classroom climate and subsequently act as a protective factor against students’ engagement in non-consensual sexting.

To sum up, the present study examined the effectiveness of a school intervention, through the TABBY program, against non-consensual sexting among middle adolescents, investigating at the same time the role of sexual preoccupation awareness in sexting behaviors. Particularly, the study examined the following:

(1) Τhe effect of the intervention on middle adolescents’ non-consensual sexting behaviors, immediately after and six months after the intervention.
(2) Τhe predictive role of middle adolescents’ sexual preoccupation in their non-consensual sexting behaviors, before, immediately after, and six months after the intervention.

Based on the literature it was expected that:

(1) Experimental group’s non-consensual sexting behaviors will be reduced immediately after and six months after the intervention, reflecting the short- and long-term effectiveness of the intervention, respectively (Hypothesis 1; Athanasiades et al., 2015; Touloupis and Athanasiades, 2022).
(2) The positive predictive relationships between experimental group’s sexual preoccupation and non-consensual sexting behaviors (namely sexual preoccupation contributes to the increase of non-consensual sexting) will be weaker immediately after and six months after the intervention (Hypothesis 2; Clancy et al., 2021).

## 2 Materials and method

### 2.1 Sample

The study consisted of 280 3rd grade^1^ junior high school students (girls: 57.5% [*Ν* = 161], *M*_age_ = 15.1, SD = 0.83). The first necessary criterion for students’ participation in the study was their non-participation in any psycho-educational program or training regarding sexting and/or sexual preoccupation before the present study. The second inclusive criterion was students’ use of any of the social media platforms (e.g., Facebook, Twitter, Instagram, TikTok, Snapchat, WhatsApp) through their own or someone else’s computer or mobile phone, regardless of the frequency of such digital devices. The students attended nine randomly selected public junior high schools of mainstream education from different regions of Athens (schools response rate: 35%). To examine possible difficulties in the completion of the questionnaire, a pilot study was conducted in 61 3rd grade junior high school students (girls: 45.9% [*Ν* = 28], *M*_age_ = 14.9, SD = 0.51). The pilot sample was not included in the total sample.

### 2.2 Questionnaire

Students responded to demographic questions about their gender (“What is your gender?”) and their age (“What is your age?”), as well as to the following two self-reported scales:

#### 2.2.1 Sexting scale

Non-consensual sexting during the last year was examined via the general framework of the subsection of “Sex and Tech” questionnaire (The National Campaign to Prevent Teen and Unplanned Pregnancy, 2008), which concerns individuals engagement in sexting. This questionnaire has been previously used in Greek adolescent students (Kamariotis, 2021). However, for the needs of the present study some expressive adaptations were made, so the questions finally reflect non-consensual sexting with written text messages or pictures/videos through the three most common behaviors: sending (4 items, such as “During the last year, have you sent written messages with sexual content to unknown/known people to make them feel bad/embarrassed?”) receiving (4 items, such as “During the last year, have you received pictures/videos with sexual content from unknown/known people that made you feel bad/embarrassed?”), and third-party forwarding (4 items, such as “During the last year, have you forwarded third parties’ pictures/videos with sexual content to made them feel bad/embarrassed?”). Questions are answered on a four-point Likert type scale (from 0 = *Never* to 3 = *More than 10 times*). Due to the expressive changes in the original version of the questionnaire, a confirmatory factor analysis with the Maximum Likelihood method was applied in the present study to test the factorial validity of the scale. The results confirmed the three factor model (sending, receiving, and third-party forwarding), which had a good fit, *χ*^2^(84, *N* = 280) = 323.114, *p* < 0.05, CFI = 0.949, TLI = 0.950, RMSEA = 0.038, SRMS = 0.044. The three factors with eigenvalue >1.0 had significant interpretive values: Factor 1 = Sending, explaining 29.05% of the total variance, Factor 2 = Receiving, explaining 20.11% of the total variance, and Factor 3 = Forwarding, explaining 14.92% of the total variance. The internal consistency indexes were satisfactory: Factor 1 (*α* = 0.846), Factor 1 (*α* = 0.818), and Factor 2 (*α* = 0.783).

#### 2.2.2 Sexual preoccupation scale

Students’ sexual preoccupation was examined with the Greek translated version (with the back-to-forth method) of the sexual preoccupation subscale of the “Sexual scale” of Snell and Papini (1989). Sexual preoccupation subscale includes 10 statements (e.g., “I think about sex all the time”) about individuals’ intense thoughts or fantasies about sex. The proposals reflect a unified factor (“Sexual preoccupation”) and they are answered on a five-point Likert scale (from 1 = *Disagree* to 5 = *Agree*). Due to the first use of the scale in the Greek context, a confirmatory factor analysis with the Maximum Likelihood method was applied in the present study. The unidimensional model was confirmed with a good fit, *χ*^2^(48, *N* = 280) = 823.087, *p* < 0.05, CFI = 0.951, TLI = 0.948, RMSEA = 0.041, SRMS = 0.046. The single factor “Sexual preoccupation” had eigenvalue >1.0 and explained 63.41% of the total variance. The internal consistency index of the factor was *α* = 0.815.

### 2.3 Procedure

Upon the approval of the study by the Greek Institute of Educational Policy (Φ15/17951/Δ1, 15/02/2022) the researcher contacted the responded schools and subsequently the students’ parents/guardians to inform them about the study. After securing parents’/guardians’ approval, the researcher visited the schools and asked the students, whose parents/guardians had consented, if they meet the participation criteria (details in subsection 2.1). Within an experimental research design, the total number of students from all the participating schools was divided into an experimental (*N* = 131) and a control group (*N* = 149). Before the intervention (1st phase), all students completed the questionnaire in the classrooms (October 2022). Afterwards, the intervention (based on the TABBY program), which was enriched with experiential activities related to students’ awareness about sexual preoccupation, was implemented to the experimental group by the teachers of the participating schools. Teachers had been previously trained for eight hours by the researcher on issues related to the prevention of sexting and the sensitization/awareness about sexual preoccupation. The intervention was applied in the classrooms and its duration was eight hours divided into four weeks (November 2022). The intervention included videos and presentations concerning sexting behaviors, its associated risks and its legal issues, a discussion about gender-based stereotypes and sexual ethical attitudes/behaviors, as well as experiential activities regarding sexual preoccupation. To examine the short- and long-term effectiveness of the intervention, students from both groups completed the same questionnaire immediately after (1st post-test / middle of December 2022) and six months after the completion of the intervention (2nd post-test / middle of June 2023). The study strictly followed the ethical rules regarding students’ and teachers’ voluntary and anonymous participation.

### 2.4 Statistical analyses

Without any missing cases, different statistical tests were performed. For each test an *a priori* power analysis of sample size was performed, using G*Power 3.1 (Faul et al., 2007), with power (1-β) of 85%, medium effect (*f* = 0.05), and an alpha error of probability *α* = 0.05. The extent of non-consensual sexting between the experimental and the control group before, immediately after (short-term effectiveness) and six months after the intervention (long-term effectiveness) was investigated through repeated measures ANOVA (Noncentrality parameter δ = 2.52, Critical t = 2.21, df = 92, Actual power: 0.85, required sample: *Ν* = 129). The bivariate relationships among the variables were explored via the Pearson (Pearson *r*) correlations (Noncentrality parameter δ = 2.29, Critical t = 2.91, df = 94, Actual power: 0.84, required sample: *Ν* = 126). Τhe predictive role of sexual preoccupation in non-consensual sexting was examined thriugh Linear Regression using the enter method (Noncentrality parameter δ = 2.24, Critical t = 2.38, df = 84, Actual power: 0.85, required sample: *Ν* = 127).

## 3 Results

### 3.1 The effectiveness of the intervention

The effectiveness of the intervention was investigated immediately after (2nd phase) and six months after its completion (3rd phase). The intervention seemed to affect statistically significantly students’ involvement in non-consensual sexting, via sending, Pillai’s Trace = 0.302, *F*(3, 277) = 4.149, *p* < 0.001, partial *η*^2^ = 0.403, receiving, Pillai’s Trace = 0.211, *F*(3, 277) = 5.837, *p* < 0.001, partial *η*^2^ = 0.454, and forwarding written messages or pictures/videos with sexual content, Pillai’s Trace = 0.449, *F*(3, 277) = 4.193, *p* < 0.001, partial *η*^2^ = 0.399. Violation of the Sphericity assumption of Mauchly’s *W* (*p* <0.05) led to Huynh-Feldt’s correction of degrees of freedom in cases of sending, *F*(2.7, 298.04) = 9.114, *p* < 0.001, partial *η*^2^ = 0.432, receiving, *F*(2.8, 301.44) = 9.503, *p* <0.001, partial *η*^2^ = 0.411, and forwarding written messages or pictures/videos with sexual content, *F*(2.7, 409.11) = 9.773, *p* <0.001, partial *η*^2^ = 0.383.

According to pairwise comparisons (applying the Bonferroni criterion, *p* < 0.017) among the three phases of the study, it was found that the three ways of engagement in non-consensual sexting (sending, receiving, and forwarding), and especially via sending and receiving written messages or pictures/videos with sexual content, were statistically significantly decreased for the experimental group (see bold Means), compared to the control group, immediately after (1st post-test) and six months after the intervention (2nd post-test) (Table 1).

**Table 1:** The effectiveness of the intervention regarding non-consensual sexting behaviors.

	Experimental group (*N* = 131)						Control group (*N* = 149)					
Sexting behaviors	Before the intervention		Immediately after the intervention		Six months after the intervention		Before the intervention		Immediately after the intervention		Six months after the intervention	
	Mean	S.D.	Mean	S.D.	Mean	S.D.	Mean	S.D.	Mean	S.D.	Mean	S.D.
Sending	1.49	0.98	**1.01**	1.43	**1.08**	1.09	1.39	1.12	1.41	2.08	1.37	1.44
Receiving	1.43	1.19	**1.03**	2.32	**1.02**	0.85	1.44	1.99	1.39	1.41	1.40	1.07
Forwarding	1.31	0.93	**1.02**	1.19	**1.04**	1.23	1.14	0.55	1.11	0.83	1.16	1.01

S.D.: standard deviation.

### 3.2 Correlations between non-consensual sexting and sexual preoccupation

The bivariate correlations among the variables involved were examined for the total sample through Pearson (Pearson *r*) correlations before (1st phase), immediately after (2nd phase) and six months after the intervention (3rd phase). During the 1st phase, sexual preoccupation was positively correlated with non-consensual sexting behaviors: sending (*r* = 0.438, *p* < 0.01), receiving (*r* = 0.432, *p* < 0.01), and forwarding written messages or pictures/videos with sexual content (*r* = 0.411, *p* < 0.01). These positive correlations between sexual preoccupation and the three non-consensual sexting behaviors (especially sending and receiving written messages or pictures/videos with sexual content) were weaker immediately after (2nd phase [sending: *r* = 0.223, *p* < 0.01, receiving: *r* = 0.235, *p* < 0.01, forwarding written messages or pictures/videos with sexual content: *r* = 0.211, *p* < 0.01]), and six months after the intervention (3rd phase [sending: *r* = 0.209, *p* < 0.05, receiving: *r* = 0.201, *p* < 0.05, forwarding written messages or pictures/videos with sexual content: *r* = 0.199, *p* < 0.05]).

### 3.3 The predictive role of sexual preoccupation in non-consensual sexting

Based on Table 2, there were found significant positive predictive relationships between students’ sexual preoccupation and their non-consensual sexting behaviors before (1st phase), immediately after (2nd phase) and six months after the intervention (3rd phase). However, according to the bold standardized regression coefficients, these positive predictive relationships between sexual preoccupation and the three non-consensual sexting behaviors (especially sending and receiving written messages or pictures/videos with sexual content) were weaker for the experimental group immediately after (2nd phase) and six months after the intervention (3rd phase).

**Table 2:** The predictive role of sex preoccupation in non-consensual sexting for experimental and control group in the three phases of the study.

Three phases of the study	Predictive factor	Sexting behaviors (with written messages or pictures/videos)	Groups	*R* ^2^	*Beta*	*t*	*p*
Before the intervention	Sex preoccupation	Sending	Experimental	0.484	0.492	5.290	0.004
			Control		0.418	4.993	0.009
		Receiving	Experimental	0.442	0.448	5.909	0.003
			Control		0.396	5.182	0.007
		Forwarding	Experimental	0.402	0.421	5.873	0.004
			Control		0.415	5.048	0.009
Immediately after the intervention	Sex preoccupation	Sending	Experimental	**0.214**	**0.211**	**2.390**	**0.011**
			Control		0.405	5.093	0.005
		Receiving	Experimental	**0.208**	**0.227**	**2.909**	**0.018**
			Control		0.409	5.182	0.009
		Forwarding	Experimental	**0.188**	**0.178**	**1.973**	**0.023**
			Control		0.403	4.848	0.009
Six months after the intervention	Sex preoccupation	Sending	Experimental	**0.189**	**0.189**	**1.920**	**0.027**
			Control		0.395	5.084	0.008
		Receiving	Experimental	**0.185**	**0.182**	**1.505**	**0.030**
			Control		0.401	4.221	0.009
		Forwarding	Experimental	**0.161**	**0.155**	**1.303**	**0.034**
			Control		0.395	4.145	0.011

*Beta*: standardized regression coefficient.

## 4 Discussion

The study examined the effectiveness of a school intervention against non-consensual sexting, according to the TABBY program, in middle adolescents, also examining the role of sexual preoccupation in their sexting behaviors. Generally, the results showed the short-term (immediately after the intervention) and the long-term effectiveness of the intervention (six months after the intervention). This is reflected in the fact that adolescents in both phases reported lower engagement in non-consensual sexting behaviors (sending, receiving, and forwarding). This finding confirms Hypothesis 1, and aligns with previous studies, which report the short- and the long-term effectiveness of interventions based on the TABBY program against similar online risk behaviors such as cyberbullying (Athanasiades et al., 2015; Touloupis and Athanasiades, 2022). Also, the present finding implies that when the generally proposed guidelines for prevention programs (e.g., lectures/presentations, audio-visual material, peer group experiential activities) (Hinduja and Patchin, 2010; Van Ouytsel et al., 2014; Bhat, 2018; Ojeda Pérez and Rey Alamillo, 2021) are adapted to specific online risk behaviors, such as sexting, can also lead to positive outcomes. However, adopting a more critical view, it could be stated that the positive results of the intervention are partially expected since the research-based TABBY program has been proved effective for related online risk behaviors such as cyberbullying. Considering that sexting is seen as a distinct online risk behavior with a particular dynamic due to its sexual element (Ringrose et al., 2012; Bianchi et al., 2019), it would be important new prevention programs exclusively designed for sexting behaviors, and not being adapted to previous programs, to be tested. In this way, clearer findings regarding the effectiveness of programs genuinely oriented toward non-consensual sexting could emerge.

Furthermore, it should be highlighted that among the three behaviors of non-consensual sexting (sending, receiving, and forwarding), sending and receiving written messages or pictures/videos with sexual content seemed to be slightly more reduced in the short-term (immediately after the intervention) and long term (six months after the intervention). This finding could reflect youth’s general tendency to engage in sexting behaviors primarily through sending and receiving sexual written or audiovisual material, compared to forwarding related material to third parties (Molla-Esparza et al., 2020). Also, it could be associated with the fact that the video utilized from the TABBY program, which was the inaugural stimulus for the subsequent experiential classroom activities, focused mainly on the roles of bullies (sending) and victims (receiving) of sexting. Therefore, it is likely that the effect of the intervention was slightly stronger for the participating students with these behaviors. Undoubtedly, future examination of the effectiveness of the present or related interventions could offer more stable findings.

Additionally, sexual preoccupation proved a positive predictor of students’ engagement in non-consensual sexting behaviors before (1st phase), immediately after (2nd phase) and six months after the intervention (3rd phase). This finding confirms Hypothesis 2 and aligns with limited studies, which report that young adults’ sexual preoccupation predisposes them positively to engage in sexting behaviors, mainly via sending or receiving written messages of pictures/videos with sexual content (Clancy et al., 2021). However, it should be underlined that the sexual preoccupation awareness classroom experiential activities, included in the intervention, emphasized the discharge of students’ feeling and thoughts about sexuality (sexual preoccupation). This may contributed to the weakening of the dynamic of sexual preoccupation as a predisposing factor towards non-consensual sexting. Subsequently, this may explains the (expected) weaker positive predictive relationships that emerged between sexual preoccupation and non-consensual sexting for the experimental group immediately after and six months after the intervention. In other words, students’ enhanced awareness about their dominant sexuality issues (sexual preoccupation awareness) during the challenging period of middle adolescence, via experiential and collaborative activities full of empathy and understanding, could weaken the influence of their sexual preoccupation on their involvement in non-consensual sexting. Therefore, it is implied that related prevention programs should not only concentrate on reducing a dysfunctional behavior (non-consensual sexting). They should also be enriched with awareness activities that weaken (sexual preoccupation) or strengthen underlying relevant psychological mechanisms (e.g., empathy, self-esteem; Touloupis and Athanasiades, 2022) associated with this behavior. Undoubtedly, this predictive pattern between sexual preoccupation and non-consensual sexting behaviors needs further examination through future related studies based on the same or a similar interventions.

Considering the specific limitations of the study (small sample size, possibly socially acceptable responses, restriction to quantitative methodology, utilization of only one predictive variable), future related studies could be proposed. Studies conducted in a larger sample of adolescents, and co-examining the predictive role of other sex-related variables (e.g., sexual self-esteem) could confirm and enrich the present findings. Also, using a mixed research method with supplementary qualitative data from students via semi-structured interviews, as in other studies (Tanrikulu, 2018; Gaffney et al., 2019), could provide more evidence that the program was effective, the specific aspects that strengthened or weakened its effectiveness, and that the results were not affected by other exogenous factors.

Nevertheless, the study offers preliminary knowledge regarding a research-based school prevention program which, although it was intended for cyberbullying, through appropriate enrichments and adaptations proved effective for non-consensual sexting among adolescents. In other words, implementing experiential school activities aimed at sexual manifestation of online risk behaviors and adolescents’ sexuality may create a sexually egalitarian and violence-free classroom climate, which could act as a protector against future risk sexual behaviors in adulthood.

## Data availability statement

The datasets presented in this article are not readily available because due to the sensitive nature of the topic of the study, participants (students, parents/guardians) were assured that the data will remain strictly in the possession of the researcher. Requests to access the datasets should be directed to t.touloupis@aegean.gr.

## Ethics statement

The study involving humans was approved by the Greek Institute of Educational Policy. The study was conducted in accordance with the local legislation and institutional requirements. Written informed consent for participation in this study was provided by the participants’ legal guardians/next of kin.

## Author contributions

TT: Writing – review & editing, Writing – original draft, Methodology, Conceptualization.

## Footnotes

^1^Students’ attendance in Greek Junior High Schools lasts three years (12–15 years old) (Ministry of Education, n.d.).
